# Force enhancement in the human vastus lateralis is muscle-length-dependent following stretch but not during stretch

**DOI:** 10.1007/s00421-020-04488-1

**Published:** 2020-09-05

**Authors:** Patrick Bakenecker, Brent J. Raiteri, Daniel Hahn

**Affiliations:** 1 Human Movement Science, Faculty of Sport Science, Ruhr University Bochum, Gesundheitscampus Nord 10, 44801 Bochum, Germany; 2 School of Human Movement and Nutrition Sciences, University of Queensland, Brisbane, Australia

**Keywords:** Eccentric, Force–length relation, Knee extensors, Muscle history dependence, Quadriceps

## Abstract

**Purpose:**

Force enhancement is the phenomenon of increased forces during (transient force enhancement; tFE) and after (residual force enhancement; rFE) eccentric muscle actions compared with fixed-end contractions. Although tFE and rFE have been observed at short and long muscle lengths, whether both are length-dependent remains unclear in vivo.

**Methods:**

We determined maximal-effort vastus lateralis (VL) force-angle relationships of eleven healthy males and selected one knee joint angle at a short and long muscle lengths where VL produced approximately the same force (85% of maximum). We then examined tFE and rFE at these two lengths during and following the same amount of knee joint rotation.

**Results:**

We found tFE at both short (11.7%, *P* = 0.017) and long (15.2%, *P* = 0.001) muscle lengths. rFE was only observed at the long (10.6%, *P* < 0.001; short: 1.3%, *P* = 0.439) muscle length. Ultrasound imaging revealed that VL muscle fascicle stretch magnitude was greater at long compared with short muscle lengths (mean difference: (tFE) 1.7 mm, (rFE) 1.9 mm, *P* ≤ 0.046), despite similar isometric VL forces across lengths (*P* ≥ 0.923). Greater fascicle stretch magnitude was likely to be due to greater preload forces at the long compared with short muscle length (*P* ≤ 0.001).

**Conclusion:**

At a similar isometric VL force capacity, tFE was not muscle-length-dependent at the lengths we tested, whereas rFE was greater at longer muscle length. We speculate that the in vivo mechanical factors affecting tFE and rFE are different and that greater stretch of a passive component is likely contributing more to rFE at longer muscle lengths.

**Electronic supplementary material:**

The online version of this article (10.1007/s00421-020-04488-1) contains supplementary material, which is available to authorized users.

## Introduction

Eccentric muscle actions are important for absorbing kinetic energy and the underlying mechanisms contributing to their unique properties have been frequently examined under in vitro conditions. One unique property is that during and following an eccentric muscle action, a muscle can produce enhanced force relative to its isometric force at the same muscle length and activation level (Edman et al. 1978; Cook and McDonagh 1995). Enhanced forces during and following an eccentric muscle action are referred to as transient force enhancement (tFE) and residual force enhancement (rFE), respectively. Both tFE and rFE have been observed across muscle structural scales from single sarcomeres (Leonard et al. 2010) and isolated animal (Abbott and Aubert 1952) and human (Pinnell et al. 2019) fibres to whole human muscles (Westing et al. 1988; Cook and McDonagh 1995) working in vivo and are, thus, known properties of skeletal muscle.

tFE and rFE are influenced by different mechanical factors. tFE is stretch-amplitude- and stretch-velocity-dependent (Edman et al. 1978; Dudley et al. 1990; Lombardi and Piazzesi 1990; Lee and Herzog 2002). However, the stretch-velocity dependence of tFE is not apparent from above ~ 0.2–0.5 times the muscle’s maximum shortening velocity in vitro (Edman et al. 1978; Harry et al. 1990; Linari et al. 2004). rFE is independent of stretch velocity (Edman et al. 1978; Tilp et al. 2009), but dependent on stretch amplitude, where rFE generally increases with increasing stretch amplitudes (Edman et al. 1978, 1982) to some critical value (Bullimore et al. 2007; Hisey et al. 2009). However, some in vivo findings suggest that rFE is only stretch-amplitude-dependent under specific circumstances, which depend on the muscles of interest and the amount of joint rotation (whereby larger joint rotation magnitudes are assumed to cause larger amplitude muscle stretches) (Lee and Herzog 2002; Oskouei and Herzog 2006; Hahn et al. 2007; Tilp et al. 2009). The discrepancy between the mechanical factors that affect in vitro and in vivo rFE might be due to both neural and mechanical factors, such as differences in how and when the muscle is activated and the amount of muscle–tendon unit (MTU) compliance, all of which may affect the amplitude and velocity of muscle stretch.

Muscle force production during and after an eccentric muscle action also depends on where the muscle operates on its force–length relationship. For in vitro and in situ studies, where muscle lengths can be well controlled, greater tFE and rFE typically occur at longer sarcomere and fascicle lengths (Granzier et al. 1989; Hisey et al. 2009; Scott et al. 1996). The few in vivo human studies that have tested the influence of muscle length on tFE and rFE show conflicting results. During in vivo multi-joint contractions, tFE has been observed at longer muscle lengths only (Hahn et al. 2014). However, during single-joint contractions, tFE has been found at both short and long muscle lengths when the preloads (i.e., the torque before the stretch starts) is similar to the angle-joint-specific maximum isometric force (Linnamo et al. 2006). In vivo studies showed that greater rFE occurs at short and long muscle lengths, but with grater rFE at long compared with short muscle lengths (Shim and Garner 2012; Power et al. 2013; Fukutani et al. 2017). Another in vivo study on the elbow flexors did not observe rFE at short or long muscle lengths (de Brito Fontana et al. 2018). The discrepancies between in vivo studies might be because of differences in the amplitude and/or velocity of muscle stretch between short and long muscle lengths, or because of differences in the muscle’s isometric force capacity between short and long muscle lengths. The discrepancies between in vivo and in vitro findings might also arise due to differences in the magnitude and velocity of muscle stretch and/or how muscle force is transmitted across the joint (Ruttiman et al. 2019) and differences in how muscles are activated (voluntary contractions versus electrical stimulation). These factors limit the ecological validity of in vitro tFE and rFE findings and suggest that more carefully controlled in vivo studies are required to assess the relevance of tFE and rFE for everyday human movement.

Due to the conflicting tFE and rFE findings at short and long in vivo muscle lengths, we sought to investigate tFE and rFE on the ascending (i.e. short muscle length) and descending limbs (i.e. long muscle length) of the estimated vastus lateralis (VL) force–angle relationship following the same amount of knee joint rotation. To ensure that tFE and rFE were not influenced by differences in the muscle’s isometric force capacity at short and long muscle lengths, we matched the VL’s isometric force capacity at the short and long muscle length. Due to the various mechanisms predicted to contribute to tFE and rFE, including (1) sarcomere length non-uniformities at longer muscle lengths (Morgan 1990), (2) inappropriate cross-bridge attachment at shorter muscle lengths (Scott et al. 1996), (3) decreased myofilament lattice spacing at longer muscle lengths (Edman 1999), and (4) increased titin forces at longer muscle lengths (Flann et al. 2011), we expected greater tFE at long compared with short muscle lengths. For these same reasons, we expected greater rFE at a long muscle length considering that enhanced forces following stretch are related to the enhanced forces during stretch (Bullimore et al. 2007; Paternoster et al. 2016).

## Methods

### Participants

Twelve healthy male subjects (age 28.4 ± 2.6 years; height 182.1 ± 4.9 cm; weight 80.8 ± 8.5 kg) gave free written informed consent prior to participating in the study. All participants were free of knee injuries and neuromuscular disorders. All experimental procedures were approved by the local Ethics Committee of the Faculty of Sport Science at Ruhr University Bochum, which conformed with the Declaration of Helsinki.

### Experimental set-up

Participants performed knee extension contractions with their right leg while sitting in a reclined position with their hip fixed at 100° of flexion on the seat of a motorized dynamometer (IsoMed2000, D&R Ferstl, GmbH, Hemau, GER). The participant’s right lower leg was fixed with Velcro around the mid-shank to a cushioned attachment that was connected to the crank arm of the dynamometer. Two shoulder restraints and one waist strap were used to secure participants firmly in the seat of the dynamometer. Participants folded their arms across their chest prior to each contraction to limit accessory movements. To compensate for the knee joint rotation that occurs during activation of the knee extensors (Arampatzis et al. 2004; Bakenecker et al. 2019), the knee joint and the dynamometer axes of rotation were aligned during a maximum voluntary contraction (MVC) at 60° knee flexion.

### Torque measurements

The dynamometer was used to measure the crank arm angle and the net knee joint torque produced during stretch, stretch–hold and fixed-end contractions. Torque and angle data were sampled at 1 kHz and synchronised using a 16-bit Power 1401 and Spike2 data collection system (Cambridge Electronic Design, UK). To later account for the effects of gravity and passive joint torque on the torque measurements, five passive knee extensions (5°s^−1^) were performed over the whole knee joint range of motion (ROM) while participants were instructed to relax and EMG signals were visually inspected by the investigator.

### Knee joint kinematics

Due to soft tissue deformation and dynamometer compliance during MVCs (Arampatzis et al. 2004), knee joint angles can differ from the crank arm angle of the dynamometer. We, therefore, determined the actual knee joint angle across contraction conditions using two inertial measurement units (IMU) (myon AG, Schwarzenberg, Switzerland) positioned on the shank and the thigh of the right leg. IMUs were secured over these segments where the least soft tissue movement occurred (i.e. on the tibia close to the knee and approximately 5 cm below the greater trochanter) using elastic straps and sports tape. Data were sampled at 143 Hz using iSen software (version 3.8 beta, STT Systems, San Sebastian, Spain) and synchronised with all other data in Spike2 software via digital pulses from the IMU and ultrasound systems.

### Surface electromyography

Surface EMG (AnEMG12, OT Bioelettronica, IT) was used to record the muscle activities of the vastus lateralis (VL), rectus femoris (RF) and vastus medialis (VM) of the right leg. After skin preparation (i.e. shaving, abrading and swabbing the skin with antiseptic), two surface electrodes (8 mm recording diameter, Ag/AgCl, Kendall H124SG, Massachusetts, USA) were placed over the muscles of interest according to SENIAM guidelines (Hermens et al. 2000) with a 2 cm inter-electrode distance. A single reference electrode was secured to the right-sided fibular head. EMG signals were band-pass filtered between 0.01 and 4.4 kHz and amplified 1000 times (AnEMG12, OT Bioelettronica, IT), before being sampled at 2 kHz using the hardware and software described above.

### Ultrasound imaging

To image the muscle fascicles of VL during stretch, stretch–hold and fixed-end contractions, a PC-based ultrasound system (LogicScan 128 CEXT-1Z Kit, Telemed, Vilnius, Lithuania) with a flat-sided 96-element transducer (LV7.5/60/96, B-mode, 8.0 MHz, 60 mm depth; Telemed, Vilnius, Lithuania) was used. The transducer captured images at ~ 62 Hz and was placed over the mid-belly of VL (Sharifnezhad et al. 2014). The location of the transducer on the skin was secured using a custom 3D-printed plastic frame and adhesive tape. The ultrasound system generated a digital pulse that was used to synchronize all digital signals to a common start and end time. VL fascicle lengths were linearly extrapolated and pennation angles were calculated relative to the horizontal in each image offline using previously described ultrasound tracking software and procedures (Gillett et al. 2013; Farris and Lichtwark 2016). During fascicle shortening, lengthening and isometric-hold phases, a frame-by-frame manual digitisation was performed if fascicle endpoints were incorrectly identified during the semi-automated tracking process.

### Experimental protocol

The entire experiment consisted of two familiarization sessions and two test sessions. Familiarization helped participants to become comfortable with the dynamometer and with performing MVCs during stretch, stretch–hold and fixed-end contractions. Each experimental session started with a standardised warm-up (walking) and five submaximal fixed-end knee extension contractions (~ 50–80% of perceived maximum effort) to precondition the MTU (Maganaris et al. 2002). Contractions were performed with standardized verbal encouragement from the investigator, while participants received real-time visual feedback of their net knee joint torque (Gandevia 2001).

### Test session 1: determination of force-angle relationship

We estimated the participants’ individual force–angle relationships to match the muscle-specific force produced by the VL at short and long muscle lengths. Participants performed maximum voluntary isometric knee extension contractions at six knee flexion angles in increments of 10° ranging from 40° to 90°. To estimate in vivo VL muscle force, the peak knee extension torque at each joint angle was multiplied by literature-based values of the VL’s physiological cross-sectional area (PCSA) relative to the quadriceps’ PCSA (34%) (Akima et al. 1995) and then divided by the angle-specific patella tendon moment arm from the recommended mean patella tendon moment arm function provided by Bakenecker et al. (2019).

### Test session 2: Determination of transient and residual force enhancement at short and long muscle lengths with a similar VL isometric force capacity

**Fig. 1:**
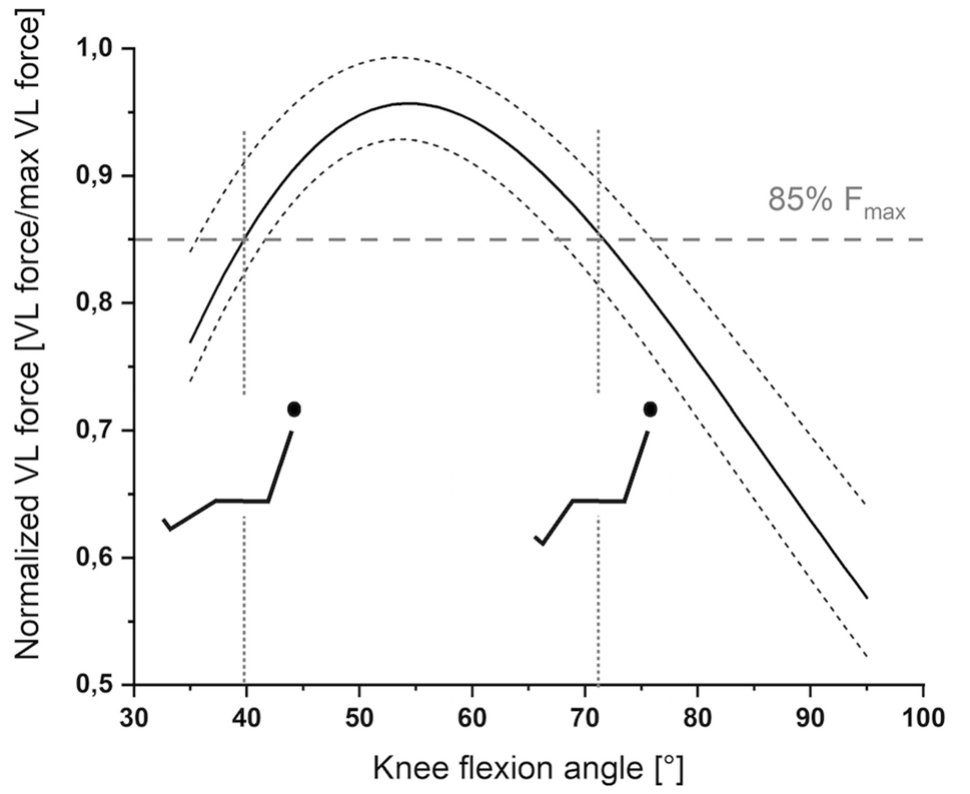
Mean normalized fitted vastus lateralis (VL) force-angle relationship (solid line) across all participants (*N* = 12) with lower and upper 95% confidence intervals (dashed lines). Two different knee joint angles (i.e. the short and long muscle lengths), where the VL produced the same estimated muscle force (85% of maximum VL force, F_max_), were selected as the target knee joint angles for the stretch, stretch–hold and fixed-end reference contractions

**Fig. 2:**
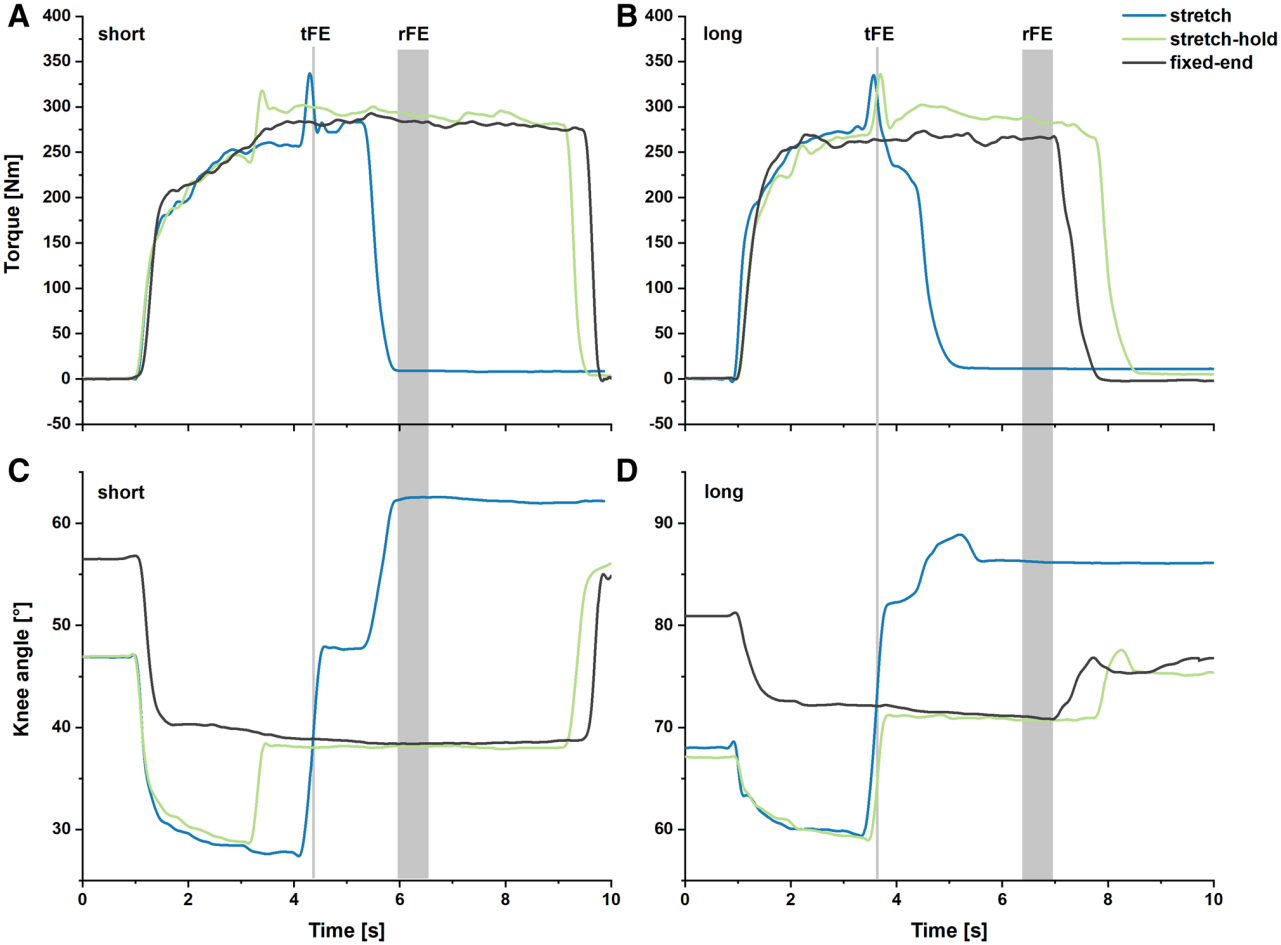
Exemplar (*n* = 1) active knee extension torque-time traces for the stretch (blue), stretch–hold (green) and fixed-end reference contractions (black) at the short (**a**) and long (**b**) muscle lengths, and the corresponding changes in knee joint angle for these three contraction conditions at short (**c**) and long (**d**) muscle lengths. The vertical grey lines and grey shaded areas show the time points/time intervals where torque was analysed to evaluate tFE and rFE

Using the estimated force–angle relationship determined in session 1, one knee joint angle on either side of the plateau of the force–angle relationship where VL produced the same estimated muscle force (85% of maximum VL force) was selected as the reference knee joint angle for the fixed-end contractions at a short and long muscle length. This reference knee joint angle was used as the target knee joint angle during stretch contractions and as the target knee joint angle following stretch in stretch–hold contractions at a short and long muscle length (see Fig. 1). Stretch contractions at short and long muscle lengths were performed with an amplitude of 25° knee flexion (15° to the target knee joint angles) to estimate transient force enhancement (tFE). Stretch–hold contractions at short and long muscle lengths were performed with an amplitude of 15° knee flexion to the final knee joint angles and followed by an isometric-hold phase to estimate residual force enhancement (rFE) (see Fig. 2). Both stretch and stretch–hold contractions were performed with an angular velocity of 60°s^−1^ and knee joint rotation was triggered manually by the investigator when participants reached a torque plateau close to their angle-specific maximum knee extension torque as determined in test session 1. All three contraction conditions (i.e., stretch, stretch–hold and fixed-end reference contractions) were performed at least three times in a randomised order and participants received at least 3 min of rest between contractions.

### Data analysis

Torque data were filtered using a dual-pass fourth-order 20 Hz low-pass Butterworth filter. Knee angle and crank arm angle data were filtered using the same filter with a 15 Hz cut-off frequency. To determine knee extension torque during the MVCs in test session 1, co-contraction was assumed to be negligible and the angle-specific torque from the fifth passive knee extension (Esteki and Mansour 1996; Lieber et al. 2000) was subtracted from the highest MVC torque value obtained at each tested knee joint position. VL fascicle length data and knee joint torque data during the passive rotations were used to check if it was necessary to account for changes in passive tension in the VL during the MVC trials, but we did not test at long enough muscle lengths to observe worthwhile changes in passive knee joint torque. Torque data obtained during MVCs in test session 1 and during stretch, stretch–hold and fixed-end reference contractions in test session 2 was subsequently used to estimate VL muscle force as described in test session 1.

#### Force–angle relationship

For each tested knee joint angle, the estimated maximum VL force was normalised to the maximum VL force over all tested knee joint angles. Following this, a second-order exponential curve was fitted to the estimated VL force–angle data.

#### Transient and residual force enhancement

tFE was calculated as the normalised difference in VL force at the respective target knee joint angle between the stretch and fixed-end reference contractions. rFE was calculated as the normalised difference in mean VL force from 2.5 to 3.0 s after stretch at the respective target knee joint angle between the stretch–hold and fixed-end reference contractions, which is in line with previous studies (Hahn et al. 2010, 2012).

#### Preload forces

VL preload forces before the start of knee joint rotation during the stretch and stretch–hold contractions were quantified at the last time point before the crank arm changed position. The start of crank arm rotation was defined as the time when its derived angular velocity was ≥ 0.3°s^−1^.

#### EMG activity

EMG signals from the superficial quadriceps muscles (VL, RF, VM) had the DC offset removed and were then smoothed using a moving root-mean-square (RMS) amplitude calculation of 100 ms. Superficial quadriceps muscle activities during the stretch contractions were then quantified as the EMG RMS value at the target knee joint angle, and for stretch–hold contractions, was quantified by taking the mean EMG RMS value from 2.5–3 s after stretch. Muscle activity during stretch and stretch–hold contractions was then compared with the time-matched muscle activity during the fixed-end contractions.

#### Fascicle behaviour

Fascicle length data were filtered using a dual-pass fourth-order 5 Hz (Bohm et al. 2018) low-pass Butterworth filter. Across contraction conditions, VL fascicle length data were analysed in the same way as the torque data to determine if fascicle lengths were similar between the stretch and fixed-end contractions and stretch–hold and fixed-end contractions. For stretch and stretch–hold contractions, the start of fascicle lengthening was determined as the time point when the VL fascicle velocity was ≥ 0.05 mms^−1^ and for the stretch–hold contractions only, the end of fascicle lengthening was determined as the time point when VL fascicle velocity was ≤ 0.05 mms^−1^. During the stretch contractions, the magnitude of fascicle stretch was measured from the time point defined above to the time point where the target joint angle was reached. For stretch–hold contractions, the magnitude of fascicle stretch was determined from the start to end of fascicle lengthening.

Fascicle shortening during the initial phase of force development was also measured across contraction conditions as the change in fascicle length from initial to minimum fascicle length. Initial fascicle length was defined as the mean value over the first 1.5 s of fascicle length data where subjects were instructed to relax and EMG signals were inspected by the investigator. To determine the stretch velocity of the VL fascicles during the stretch and stretch–hold contractions, the filtered fascicle length data were differentiated. Stretch velocity during the stretch contractions was then defined as the VL fascicle velocity at the time point where the target joint angle was reached. Stretch velocity during the stretch–hold contractions was defined as the mean VL fascicle velocity over the period of fascicle lengthening.

For six participants (the reduced sample size was due to time drift in the IMU signals during stretch and stretch–hold contractions), internal work of the VL muscle fascicles was also calculated by integrating the estimated VL fascicle force over the fascicle length change during the initial phase of force development. VL fascicle force was calculated by dividing the VL muscle force by the cosine of the measured fascicle angle, which was quantified relative to the deep aponeurosis of VL.

### Statistical analysis

All data processing and analysis were performed using custom-written scripts in Matlab software (Mathworks, R2016b, Natick, MA). Based on the calculations provided by Leys et al. (2013), one participant was defined as an outlier and removed from statistical analysis. Outliers were defined as values exceeding three times the median absolute deviation. Data were tested for normality using Shapiro–Wilk normality tests. Two-way repeated-measures ANOVAs were performed to identify differences in VL force, superficial quadriceps muscle activities and VL fascicle lengths between stretch, stretch–hold and fixed-end contractions between muscle lengths (contraction condition × muscle length). The same test was used to identify differences in VL fascicle shortening and VL fascicle work between stretch, stretch–hold and fixed-end contractions between muscle lengths. If a contraction condition main effect or significant interaction was observed, post hoc comparisons with Sidak adjustments were performed. Two-way repeated-measures ANOVAs were also used to identify differences in VL fascicle stretch, VL preload force and VL fascicle stretch velocity during stretch and stretch–hold contractions across muscle lengths. Paired *t* tests were used to identify differences in tFE and rFE between the short and long muscle lengths. Pearson or Spearman (if normality was violated) correlation coefficients were calculated to test the strength of the relationships between tFE and rFE, between tFE/rFE and fascicle stretch magnitude and between VL preload force and fascicle stretch magnitude across both muscle lengths (i.e., pooled data). The alpha level was set at *P* < 0.05 and statistical analyses were performed using commercially available software (GraphPad Prism 8, San Diego, CA, USA).

## Results

### Transient force enhancement

**Fig. 3:**
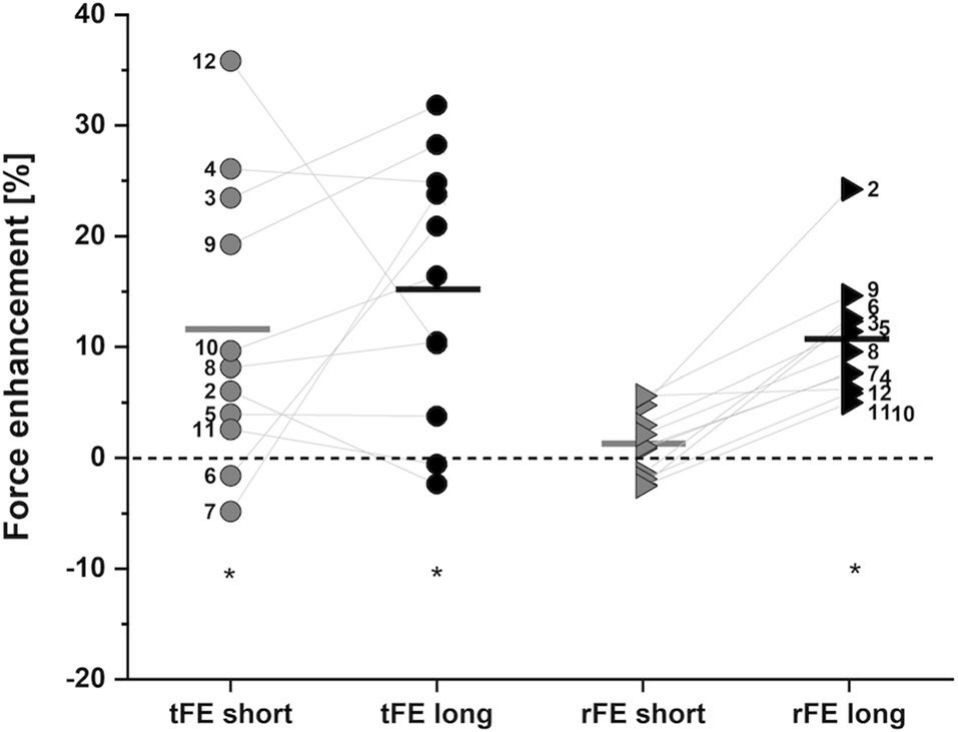
Individual and mean transient force enhancement (tFE; dots) and residual force enhancement (rFE; triangles) magnitudes at the short (grey) and long (black) muscle lengths. Filled symbols represent the individual values and horizontal lines indicate the group mean for each condition (*N* = 11). Grey lines and black numbers distinguish between participants. *Indicates a significant difference compared with time-matched fixed-end conditions at the same muscle length (*P* < 0.05)

Mean time-matched VL force during the fixed-end reference contractions was 1910 ± 382 N at the short muscle length and 1931 ± 408 N at the long muscle length, which was not significantly different (mean difference 3.1 ± 2.4%; *P* = 0.567). Mean VL force during the stretch condition was 2112 ± 348 N at the short muscle length and 2228 ± 548 N at the long muscle length. This resulted in tFE of 11.6 ± 12.8% at the short muscle length (*P* = 0.03) and 15.2 ± 11.8% at the long muscle length (*P* = 0.003) (Fig. 3), which was not significantly different between muscle lengths (*P* = 0.438). The corresponding net knee joint torques are shown in Table 1.

**Table 1:** Mean values and standard deviations for knee extension torques, VL forces and EMG amplitudes of the VL, RF and VM during the stretch and stretch–hold contractions compared with the time-matched fixed-end contractions at the short and long muscle length

Muscle length	Stretch contractions				Stretch–hold contractions			
	Short		Long		Short		Long	
	Stretch	Fixed-end	Stretch	Fixed-end	Stretch–hold	Fixed-end	Stretch–hold	Fixed-end
Knee extension torque [Nm]	313.2 ± 56.4	291.3 ± 68.6	309.8 ± 98.0*	267.6 ± 72.7	291.9 ± 76.4	284.9 ± 81.0	286.9 ± 67.4*	262.1 ± 67.3
VL force [N]	2112 ± 348*	1910 ± 382	2228 ± 548*	1931 ± 408	1957 ± 398	1934 ± 404	2066 ± 386*	1877 ± 397
VL EMG amplitude [V]	0.37 ± 0.15	0.37 ± 0.16	0.34 ± 0.17	0.36 ± 0.18	0.35 ± 0.16	0.31 ± 0.13	0.40 ± 0.19	0.37 ± 0.19
RF EMG amplitude [V]	0.31 ± 0.22	0.43 ± 0.38	0.37 ± 0.31	0.39 ± 0.39	0.44 ± 0.42	0.39 ± 0.39	0.43 ± 0.31	0.44 ± 0.35
VM EMG amplitude [V]	0.48 ± 0.28	0.54 ± 0.42	0.50 ± 0.33	0.52 ± 0.34	0.48 ± 0.37	0.46 ± 0.43	0.59 ± 0.41	0.55 ± 0.38

^*^Significantly different to fixed-end condition at *P* < 0.05

### Residual force enhancement

Mean time-matched VL force during the fixed-end reference contractions was 1934 ± 404 N at the short muscle length and 1877 ± 396 N at the long muscle length, which was not significantly different (mean difference 5.2 ± 3.2%; *P* = 0.143). Mean VL force during the stretch–hold condition was 1957 ± 398 N at the short muscle length and 2066 ± 386 N at the long muscle length. This resulted in rFE of 1.3 ± 3.1% at the short muscle length (*P* = 0.366) and 10.7 ± 5.5% at the long muscle length (Fig. 3) (*P* < 0.001), which was significantly different between muscle lengths (*P* < 0.001). The corresponding net knee joint torques are shown in Table 1. Across both muscle lengths, we found no significant correlation between tFE and rFE (*r* = 0.18, 95% CI: -0.26 to 0.56, *P* = 0.428).

### Preload forces

Mean VL preload force during the stretch contractions was 1476 ± 270 N at the short muscle length and 2082 ± 536 N at the long muscle length (mean difference 41.3 ± 23.4%), which was significantly different between muscle lengths (*P* < 0.001). VL preload force during stretch–hold contractions was 1439 ± 333 N at the short muscle length and 2100 ± 450 N at the long muscle length (mean difference 48.9 ± 29.6%), which was also significantly different between muscle lengths (*P* < 0.001). There were no significant differences in preload force between stretch and stretch–hold contractions at short (*P* = 0.972) or long muscle lengths (*P* = 0.994).

### Muscle activity

There were no significant differences in muscle activity for any of the superficial quadriceps muscles (VL, RF and VM) for the stretch condition compared with the time-matched fixed-end condition at the short (VL: *P* = 0.975; RF: *P* = 0.314; VM: *P* = 0.551) or long (VL: *P* = 0.792; RF: *P* = 0.636; VM: *P* = 0.777) muscle lengths. We also found no significant differences in muscle activity of the VL, RF and VM for the stretch–hold condition compared with the time-matched fixed-end condition at the short (VL: *P* = 0.382; RF: *P* = 0.291; VM: *P* = 0.958) or long (VL: *P* = 0.271; RF: *P* = 0.861; VM: *P* = 0.097) muscle lengths. Detailed results are shown in Table 1.

### Fascicle behaviour

**Fig. 4:**
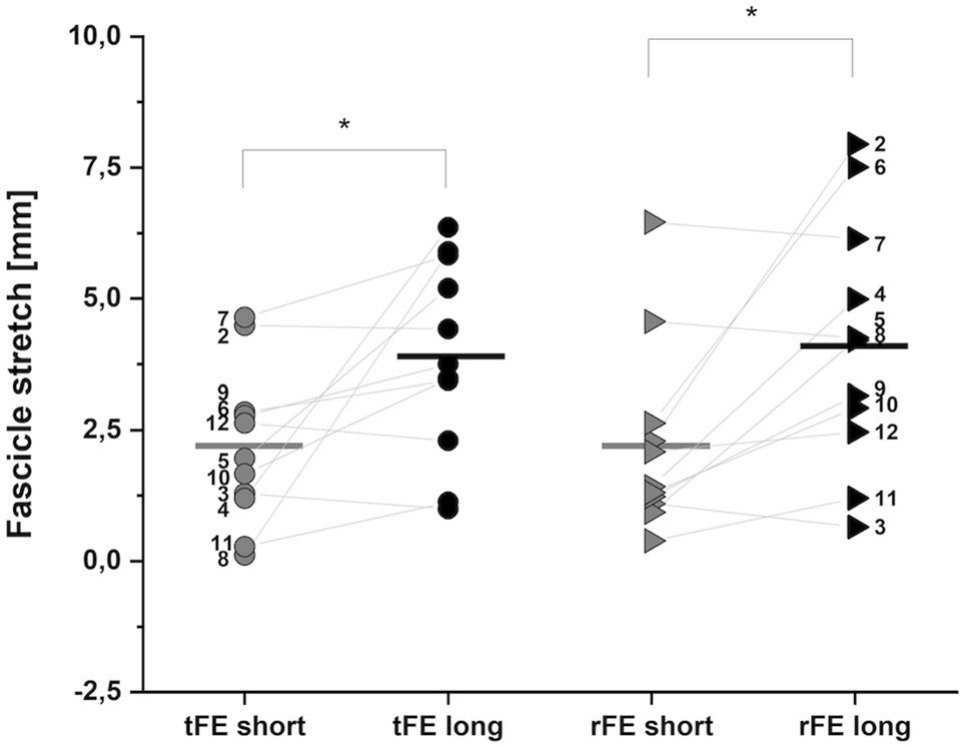
Individual and mean magnitudes of VL muscle fascicle stretch during the stretch (tFE; dots) and stretch–hold contractions (rFE; triangles) at the short (grey) and long (black) muscle lengths. Filled symbols represent the individual values and horizontal lines indicate the group mean for each condition (*N* = 11). Grey lines and black numbers distinguish between participants. *Indicates a significant difference between the short and long muscle length (*P* < 0.05)

During the stretch contractions, VL muscle fascicles were stretched 2.2 ± 1.5 mm at the short muscle length compared with 3.9 ± 1.9 mm at the long muscle length, which was significantly different between muscle lengths (*P* = 0.046). For stretch–hold contractions, VL muscle fascicles were stretched by 2.2 ± 1.8 mm at the short muscle length and 4.1 ± 2.4 mm at the long muscle length, which was also significantly different between muscle lengths (*P* = 0.016) (Table 2 and Fig. 4). A comparison of VL fascicle shortening at short and long muscle lengths between stretch, stretch–hold and fixed-end contractions during the initial phase of force development revealed no significant main effect of contraction condition (*P* = 0.952), a significant main effect of muscle length (*P* = 0.047) and no significant interaction (*P* = 0.550) (Table 2). Post hoc comparisons revealed no significant differences in VL fascicle shortening between muscle lengths during stretch (*P* = 0.416), stretch–hold (*P* = 0.891) or fixed-end (*P* = 0.292) contractions.

**Table 2:** Mean values and standard deviations for VL muscle fascicle stretch, shortening and work magnitudes and stretch velocities during the stretch and stretch–hold contractions

Muscle length	Short			Long		
Contraction condition	Stretch	Stretch–hold	Fixed-end	Stretch	Stretch–hold	Fixed-end
VL fascicle stretch [mm]	2.2 ± 1.5*	2.2 ± 1.8*	–	3.9 ± 1.9	4.1 ± 2.4	–
VL stretch velocity [mms^−1^]	0.19 ± 0.11*	0.16 ± 0.11	–	0.28 ± 0.14	0.25 ± 0.14	–
VL fascicle shortening [mm]	17.4 ± 7.7	16.4 ± 6.0	17.7 ± 5.2	15.3 ± 6.3	15.5 ± 6.1	14.4 ± 4.4
Fascicle work (*n* = 6) [J]	0.47 ± 0.30	0.29 ± 0.19	0.33 ± 0.12	0.33 ± 0.13	0.39 ± 0.16	0.31 ± 0.15

Muscle fascicle shortening and work magnitudes were determined during the initial phase of force development (i.e. preload)

^*^Significantly different to the stretch/stretch–hold condition at the long muscle length at *P* < 0.05

**Fig. 5:**
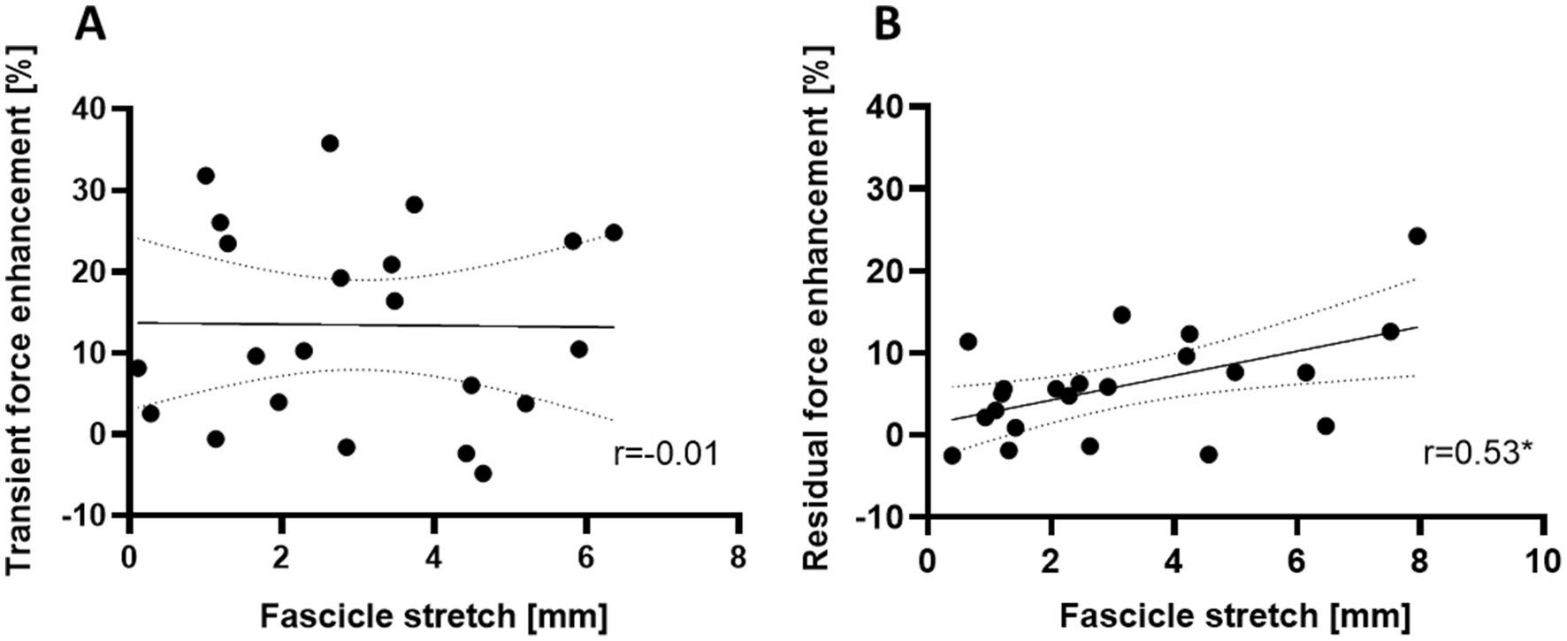
Relationship between transient (**a**) or residual (**b**) force enhancement and corresponding VL muscle fascicle stretch across both muscle lengths. *Indicates a significant relationship (*P* = 0.012; 95% CI 0.13–0.78)

Across muscle lengths, there were no significant differences in absolute VL fascicle lengths between stretch (*P* = 0.610) or stretch–hold (*P* = 0.074) contractions and the fixed-end reference contractions. We found significantly lower VL fascicle stretch velocities during stretch contractions (*P* = 0.026) at the short compared with the long muscle length, but no significant difference in VL fascicle stretch velocity was observed during stretch–hold contractions (*P* = 0.067) between the short and long muscle length (Table 2). Across muscle lengths, we found no significant correlations between VL fascicle stretch magnitude and tFE (*r* = − 0.01, 95% CI − 0.43 to 0.41, *P* = 0.956) and between VL fascicle stretch velocity and tFE (*r* = 0.03, 95% CI − 0.39 to 0.45, *P* = 0.880). However, there were significant moderate positive correlations between VL fascicle stretch magnitude and rFE (*r* = 0.53, 95% CI 0.13–0.78, *P* = 0.012) (Fig. 5) and between VL preload force and VL fascicle stretch magnitude across muscle lengths (rho = 0.51, 95% CI 0.24–0.70, *P* < 0.001).

### Kinematics

The mean knee joint angle determined from the IMUs for the fixed-end reference contractions was 39.8 ± 2.9° and 69.3 ± 9.4° for the short and long muscle lengths, respectively. The contraction-induced knee joint rotation (despite fixed-end conditions) was 19.2 ± 4.0° and 15.3 ± 2.9° at the short and long muscle lengths, respectively. During the preload phase of the stretch and stretch–hold contractions at the short muscle lengths, the knee joint rotated 17.8 ± 3.8° and 16.2 ± 4.2°, respectively. During the preload phase of the stretch and stretch–hold contractions at the long muscle lengths, the knee joint rotated 17.3 ± 5.6° and 17.1 ± 2.2°, respectively.

## Discussion

The main purpose of this study was to investigate if tFE and rFE differs following the same knee joint rotation magnitude at short and long muscle lengths with the same estimated isometric VL force capacity. We found that VL force during stretch contractions (tFE) was significantly larger than the time-matched VL force during fixed-end contractions at both short and long muscle lengths and that there was no significant difference in tFE between muscle lengths. In contrast, VL force during stretch–hold contractions (rFE) was only significantly larger than the time-matched VL force during fixed-end contractions at the long muscle length, resulting in significantly greater rFE at the long compared with short muscle length. Therefore, at the muscle lengths tested here, rFE, but not tFE, was muscle-length-dependent, which suggests that the in vivo mechanical factors contributing to tFE and rFE are different.

In this study, we observed VL forces that were enhanced by 12% at a short muscle length and by 15% at a long muscle length during stretch contractions relative to maximum-effort fixed-end contractions at matched target joint angles. This is in accordance with other in vivo studies on the knee extensors, which reported enhanced forces ranging 10–30% during maximum-effort stretch contractions relative to corresponding fixed-end reference contractions (Finni et al. 2003; Hahn et al. 2007, 2010). Similar to our study, these studies incorporated participant familiarisation into their experimental design and ensured high preloads prior to joint rotation, which are crucial factors when investigating tFE and rFE (Hahn 2018). Other studies that have not observed enhanced forces during stretch contractions at short or long muscle lengths (Westing et al. 1988; Komi et al. 2000; Doguet et al. 2017) may be confounded by inadequate participant familiarisation and/or insufficient preloads prior to joint rotation. For example, in vivo tFE may not be studied if there are low preloads before joint rotation, which can result in concentric and not eccentric fascicle behaviour during MTU stretch due to increased MTU compliance at low muscle forces (Hahn 2018). This argument is supported by findings from Linnamo et al. (2006), who only observed tFE in participants who reached preload forces within ± 5% of their angle-specific maximum isometric force before joint rotation. Our results further confirm that concentric muscle fascicle behaviour of the VL during the active MTU lengthening phase can be completely avoided with high preloads (Fig. 2).

### Transient force enhancement

In vitro studies have previously revealed two mechanisms that are associated with enhanced forces during stretch (tFE) contractions: increased cross-bridge forces (Huxley and Simmons 1971) and increased passive forces caused by stretch of the spring-like muscle protein titin (Granzier and Labeit 2006). Regarding a length dependency of these mechanisms, it has been shown that there is inappropriate cross-bridge attachment at shorter muscle lengths (Scott et al. 1996), decreased myofilament lattice spacing at longer muscle lengths (Edman 1999) and increased titin forces at longer muscle lengths (Flann et al. 2011). Therefore, tFE during stretch contractions is expected to be larger when the muscle is stretched at longer lengths. However, our results show no significant difference in tFE between the muscle lengths we tested (Fig. 3), with only a 3.6% MVC mean difference between short and long muscle lengths, which suggests that tFE is not muscle-length-dependent in the knee extensors over the muscle length range we tested here.

Our tFE findings contradict those from in vitro and in situ studies, which suggest tFE increases with increasing muscle length (Granzier et al. 1989; Scott et al. 1996). The discrepancy could be due to differences in how the muscle is activated during in vitro and in vivo experiments. Some studies (Westing et al. 1988; Dudley et al. 1990; Webber and Kriellaars 1997; Babault et al. 2001) suggest that the absence of tFE during in vivo stretch contractions arises due to neural inhibition and that neural inhibition might be greater at longer muscle lengths (Linnamo et al. 2006). These studies defined neural inhibition as the difference in maximal torque produced during electrical stimulation compared with voluntary contraction. Although we did not assess neural inhibition in this way, neural inhibition might have reduced tFE at the long muscle length, resulting in no difference in tFE between muscle lengths. However, our EMG data show no significant differences in muscle activity of VL, RF and VM during stretch contractions compared with the corresponding fixed-end contractions at both muscle lengths, and knee extension torques and VL muscle forces during stretch were similar across muscle lengths. This suggests either that neural inhibition was not present, that neural inhibition was similar between muscle lengths because of similar knee extension torques and muscle forces, or that surface EMG measurements are not sensitive enough to detect differences in neural inhibition. Although our data suggest that the in vitro muscle-length dependency of tFE is not relevant in vivo under maximum voluntary effort, we did not test at VL fascicle lengths much longer than optimal and so we cannot exclude this possibility based on our investigation. Testing at comparably long muscle lengths to in vitro studies was not possible here due to the constraints of the dynamometer.

In vitro (Edman et al. 1978; Lombardi and Piazzesi 1990) and in vivo (Dudley et al. 1990; Lee and Herzog 2002) studies have shown that tFE increases with increasing stretch magnitudes and stretch velocities. In our experiment, we found significantly greater VL fascicle stretch magnitudes and velocities at the long compared with the short muscle length. However, the greater mean VL fascicle stretch magnitude and velocity did not result in greater mean tFE at the long compared with the short muscle length. We also did not observe a significant positive correlation between tFE and fascicle stretch magnitude across muscle lengths, so we speculate that the difference in fascicle stretch magnitude across muscle lengths was not large enough to cause significantly greater tFE at the long compared with the short muscle length. The greater mean VL fascicle stretch velocity at the long muscle length likely did not affect the mean tFE across muscle lengths because the muscle fascicles were being stretched on the plateau region of their eccentric force–velocity relationship. Even though the observed tFE difference between short and long muscle lengths was not significant, the mean tFE at the long muscle length was greater than that at the short muscle length by ~ 4%. Further, tFE showed substantial variability (Fig. 3). Accordingly, we might be underpowered to detect a significant difference in tFE between lengths.

### Residual force enhancement

We observed only small and non-significant rFE (1.3 ± 3.1%) at the short muscle length, but greater and significant rFE (10.6 ± 5.5%) at the long muscle length. Hence, rFE was muscle-length-dependent across the length range we tested here. Similar in vivo results from the knee extensors have previously been reported (Shim and Garner 2012). However, this is not consistent across all studies, as Power et al. (2013) found significant rFE in the knee extensors at short and long muscle lengths. The contrasting findings at the short muscle length might be due to the greater stretch amplitude (60° knee joint rotation) in the former study and/or participants acting closer to the plateau of their force–length relationship (60° target joint angle for the short muscle length) compared with our study. Similar to our findings though, Power et al. (2013) did observe greater rFE at the longer muscle length. Experiments on other lower limb extensors (plantar flexors) show similar muscle-length-dependent results for rFE on the ascending limb and plateau region of the force–length relationship. Findings from Pinniger and Cresswell (2007), Hahn et al. (2012), Hahn and Riedel (2018) and Fukutani et al. (2017) indicate that rFE of ~ 5–17% can be observed when stretches are applied at longer muscle lengths (i.e., at from ankle joint angles of − 5° to 20° dorsiflexion), whereas stretches over the same amplitude starting at a shorter muscle length (i.e., from − 15° to 0° dorsiflexion) do not result in significant rFE.

From an in vitro perspective, the differences in rFE at the short and long muscle lengths could be primarily attributed to non-crossbridge factors. This is because regarding the sarcomere length non-uniformity theory (Julian and Morgan 1979), (1) instabilities are only evident at long muscle lengths, but rFE has also been observed on the ascending limb and plateau region of the force–length relationship and (2) sarcomere length non-uniformities are not different during fixed-end contractions and the steady-state hold phase of stretch–hold contractions (Johnston et al. 2019). Besides cross-bridge mechanisms and sarcomere length non-uniformities, the latest research has found evidence supporting the idea that the engagement of a passive structural element (titin) can explain enhanced forces after active muscle stretch (Herzog et al. 2016; Shalabi et al. 2017; Herzog 2018). Our in vivo rFE results are supported by findings from Rassier and Herzog (2005), who observed an increase in titin stiffness with increasing muscle length. Therefore, stretches at longer muscle lengths could lead to greater rFE due to an increase in titin stiffness. Moreover, greater rFEs at longer muscle lengths are further supported by findings from Nocella et al. (2014) and Morgan et al. (2000), who showed that rFE was smaller or non-existent when stretch began on the ascending limb compared with the plateau region or descending limb of the force–length relationship.

From an in vivo perspective, predictions about mechanisms, such as titin engagement, during stretch and stretch–hold contractions become difficult because muscles act in series with long tendinous tissues within their MTU and are activated in concert with synergist and antagonist muscles. Regarding MTU compliance in vivo, Ichinose et al. (1997) reported that it decreases with increasing muscle length, where the stiffness of the MTU was estimated as the change in VL fascicle length over a given estimated change in patella tendon force. In our study, we only matched VL forces at two muscle lengths and we expected similar VL muscle fascicle stretch magnitudes across muscle lengths. As we found significantly more VL fascicle stretch during stretch–hold contractions at long compared with short lengths (Fig. 4), despite identical joint rotations, greater MTU compliance at short muscle lengths might be responsible. This is supported by our data as we found that preload forces account for 26% of the variance in VL fascicle stretch magnitudes across muscle lengths, with greater preloads increasing the amount of fascicle stretch, presumably by reducing MTU compliance and reducing the tendinous tissues’ ability to buffer active muscle lengthening during MTU lengthening (Konow and Roberts 2015). Thus, preload forces and forces during stretch should be considered in future studies that attempt to match muscle fascicle stretch amplitudes when assessing rFE at different muscle lengths in vivo.

Our finding of no rFE at the short muscle length could be influenced by four non-responders during the stretch–hold contractions (Fig. 3). The non-responder phenomenon was initially described by Oskouei and Herzog (2006) and has been confirmed by others (Hahn et al. 2007, 2012; Tilp et al. 2009). Two of the four non-responders in this study showed reduced VL fascicle stretch compared with the group mean and three of the non-responders showed lower muscle activity levels in the VL, RF or VM compared with the corresponding fixed-end contractions. However, all non-responders at short muscle lengths showed rFE at long muscle lengths, which suggests that reduced net knee extensor muscle activity and less VL fascicle stretch might explain why they were non-responders (i.e. no rFE) only at the short muscle length.

### Residual force depression

Despite our motivation to investigate tFE and rFE during stretch and stretch–hold contractions with a maximum preload, our VL fascicle data show shortening–stretch and shortening–stretch–hold behaviours, respectively. rFE has been shown to be influenced in a dose-dependent manner by the amount of shortening preceding stretch (Lee et al. 2001) and active shortening is known to cause a long-lasting reduction in steady-state isometric force following shortening [i.e. residual force depression (Abbott and Aubert 1952; Granzier and Pollack 1989)], which can affect dorsiflexion force during fixed-end contractions (Raiteri and Hahn 2019). Consequently, we investigated fascicle behaviour during the initial phase of force development for each contraction condition. From six participants, we calculated fascicle shortening magnitudes and estimated fascicle shortening work over this period. We found no significant differences in either VL fascicle shortening magnitude or fascicle shortening work across contraction conditions at the same muscle length or across muscle lengths for the same contraction condition (Table 2). Therefore, potential shortening-induced residual force depression did not appear to confound our tFE and rFE findings across muscle lengths.

### Relevance

Our in vivo results conflict with previous in vitro findings regarding the stretch-amplitude dependence of tFE. Although in vivo human studies have less potential to contribute to a detailed understanding of rFE-related mechanisms, from an applied perspective they can offer new insights into the ‘everyday’ physiological relevance of tFE and rFE during voluntary human movement (Seiberl et al. 2013; Paternoster et al. 2016). Eccentric muscle actions of the lower extremity are particularly important for absorbing kinetic energy during landing tasks and recently an increased contribution of the knee and hip joints to energy absorption during human hopping was shown following higher perturbation heights (Dick et al. 2019). Greater knee joint flexion following higher drops may increase the potential of tFE and rFE to help humans stabilise fall recovery following unexpected perturbations, as well as during expected drops (Hollville et al. 2019). Therefore, in vivo experiments investigating the mechanical factors that influence tFE and rFE are needed to better understand how these phenomena contribute to everyday muscle function and potentially enhance muscle performance and improve movement economy during energy-absorbing tasks.

### Limitations

The approach we used to estimate VL muscle forces neglects changes in muscle shape, muscle architecture (e.g. pennation angle), and contributions from antagonistic muscles, such as the hamstrings, to the net joint torque. Hamstring muscle activity could not be recorded as participants were seated during the contractions. We also assumed fixed force contributions of the individual knee extensor muscles at the short and long muscle lengths based on literature-derived PCSAs, which are likely to vary between participants due to age, gender and their level of training experience. However, as tFE or rFE values were compared across muscle lengths from the same participant in this study and surface EMG revealed similar quadriceps muscle activities, we do not believe that these limitations would systematically bias our results.

### Conclusion

The purpose of this study was to investigate whether in vivo tFE and rFE differ at short and long muscle lengths with matched isometric VL force capacities following identical knee joint rotations. We found that tFE did not significantly differ between short and long muscle lengths, despite greater fascicle stretch at the longer muscle length (mean difference: 1.8 ± 2.0 mm), which suggests that tFE is either insensitive to this difference in stretch amplitude or that neural inhibition reduces the magnitude of the tFE at longer muscle lengths in vivo. We only observed rFE at the long, but not short, muscle length, which could be masked by lower preload forces at the short muscle length resulting in significantly less VL muscle fascicle stretch compared with the long muscle length. Thus, the difference in fascicle stretch (mean difference: 2.1 ± 2.1 mm) that we observed might contribute to our observation of rFE being muscle-length-dependent. Differences in the amount of VL fascicle shortening and work prior to fascicle stretch do not help to explain the differences in rFE we found between the short and long muscle lengths. Future studies that attempt to investigate how muscle length and stretch amplitude affect tFE and rFE in vivo should consider testing over a greater length range in a different muscle group (e.g. at lengths with and without substantial passive muscle force) and matching preload forces and forces during stretch [e.g. through a simple shear-wave tensiometer (Martin et al. 2018)], as well as assessing individual force contributions from synergist muscles to tFE and rFE during submaximal voluntary contractions [e.g. through supersonic shear-wave imaging (Bouillard et al. 2011)].

## Electronic supplementary material

Below is the link to the electronic supplementary material.

Additional file1 (DOCX 13 kb)

## Abbreviations

ANOVA: Analysis of variance
EMG: Electromyography
IMU: Inertial measurement unit
MTU: Muscle–tendon unit
MVC: Maximal voluntary contraction
PCSA: Physiological cross-sectional area
RF: Rectus femoris
rFE: Residual force enhancement
RMS: Root-mean-square
ROM: Range of motion
tFE: Transient force enhancement
VL: Vastus lateralis
VM: Vastus medialis
