# Supplementary material for: Force enhancement in the human vastus lateralis is muscle-length-dependent following stretch but not during stretch
Source: Eur J Appl Physiol. 2020 Sep 5;120(12):2597–610. doi: 10.1007/s00421-020-04488-1 (PMC7674334; doi:10.1007/s00421-020-04488-1)
Supplement: Supplementary file 1 — Additional file1 (DOCX 13 kb) [file 421_2020_4488_MOESM1_ESM.docx]

Table 3. Mean values and standard deviations for absolute VL fascicle lengths during the stretch and stretch-hold contractions compared with the time-matched fixed-end contractions at the short and long muscle lengths.

|  |  | Absolute VL fascicle length [mm] | | | | |
| --- | --- | --- | --- | --- | --- | --- |
| Muscle length/  contraction condition |  | stretch | fixed-end |  | stretch-hold | fixed-end |
| short |  | 104.0 ± 18.1 | 104.4 ± 18.8 |  | 103.5 ± 18.9 | 104.2 ± 18.6 |
| long |  | 125.7 ± 18.9 | 126.0 ± 19.1 |  | 125.0 ± 18.7 | 126.1 ± 19.8 |
